# Lipid-Lowering Effects of Carob Extracts (*Ceratonia siliqua*): Proposed Mechanisms and Clinical Importance

**DOI:** 10.3389/fphar.2022.921123

**Published:** 2022-06-29

**Authors:** Marko Nemet, Milica Vasilić, Ana Tomas

**Affiliations:** ^1^ Faculty of Medicine, University of Novi Sad, Novi Sad, Serbia; ^2^ Department of Pharmacology, Toxicology and Clinical Pharmacology, Faculty of Medicine, University of Novi Sad, Novi Sad, Serbia

**Keywords:** carob, fiber, polyphenols, dyslipidemia, cholesterol, triglycerides

## Abstract

The global prevalence of dyslipidemia (elevated plasma levels of total cholesterol, LDL-Cholesterol, triglycerides, and lower plasma levels of HDL-Cholesterol) is constantly on the rise. Lately, carob pulp has been recognized as an effective natural product for the treatment of dyslipidemia. The two main components of the carob pulp, polyphenols, and insoluble fiber are believed to have beneficial effects on lipid metabolism. Studies on humans and animals confirmed its lipid-lowering effects. Several mechanisms have been proposed to explain this phenomenon, namely by affecting three organ systems: 1) gastrointestinal tract, 2) liver and 3) adipose tissue. Also, carob products have antioxidative, anti-inflammatory, and vascular-protective activity.

## Introduction

The global prevalence of dyslipidemia [elevated plasma levels of total cholesterol, LDL-cholesterol, triglycerides (TAG), and lower plasma levels of HDL-cholesterol] is constantly on the rise. Dyslipidemias are classified as primary, secondary, or dyslipidemias due to a combination of genetic factors and a triggering event. Primary dyslipidemias, also called familial dyslipidemias, are genetic disorders (Hachem and Mooradian, 2006). Secondary dyslipidemia is caused by poor eating habits, sedentary lifestyles, life habits, various medical conditions, and medication. Important diseases that can present with altered lipid levels are hypothyroidism, nephrotic syndrome, primary biliary cholangitis, diabetes, and Cushing’s syndrome. The use of thiazides, beta-blockers, corticosteroids, cyclosporine, protease inhibitors, steroid hormones, and atypical antipsychotics has also been shown to be associated with lipid level disturbances. Alcohol and tobacco consumption have also been linked to abnormal lipid status (Yanai and Yoshida, 2021). Dyslipidemia plays a major role in the pathogenesis of atherosclerosis (Falk, 2006). Therefore, it was estimated that in 2019 there were around 4 million deaths globally secondary to elevated blood LDL-cholesterol levels (Pirillo et al., 2021). Consequently, one of the key tasks of modern medicine is to combat this problem which is primarily done by lifestyle modification and the use of lipid-lowering drugs, including statins, a mainstay in the treatment of hyperlipidemia, fibrates, PCSK9 inhibitors, bile acid sequestrants, and ezetimibe (Nies et al., 2006; Grundy et al., 2019). Despite the number of conventional drugs available several factors might make long-term pharmacological treatment of dyslipidemia difficult. Namely, financial burden and fear of side effects which lead to poor adherence to conventional treatment (Sirtori, 2014; Okopien et al., 2018). Together with variable individual responses, there is an avenue for alternative management strategies, where herbal products base drugs represent a promising source of substances for the prevention and the treatment of lipid disorders.

Among a myriad of plants with potential beneficial effects on lipid level regulation, carob has been lately recognized as an effective option. The carob tree (*Ceratonia siliqua*), found all around the world, originates from the Mediterranean region. Estimated 315,000 tons of carob are produced annually, the majority originating from Spain. The two main components of the carob fruit are the seeds and the pulp (alternatively called a pod) (Stavrou et al., 2018). Carob pulp is rich in many phytochemicals such as insoluble fiber, and polyphenols, alongside cyclitols, amino acids, minerals, and vitamins (Zhu et al., 2019). Tannins, gallic acid, catechins, myricetin, quercetin, flavonoids, and their derivatives are the polyphenolic compounds that are found in carob (Stavrou et al., 2018) while the insoluble fiber consists of cellulose and hemicellulose (Rico et al., 2019). The two key components that are believed to have beneficial effects on plasma lipid levels are polyphenols and insoluble fiber (Zhu et al., 2019). The plethora of potentially beneficial phytochemicals in carob make it a promising product, but the feasibility of using carob products in the prevention or treatment of lipid disorders remains to be elucidated. Therefore, to identify and critically appraise the available evidence on carob’s potential lipid-lowering effects, PubMed and Google Scholar databases were searched using the keywords “carob,” “carob fiber,” “dyslipidemia,” “hypercholesterolemia,” “hypertriglyceridemia,” and “lipids” with no time constraint. Potential studies were analyzed initially by the title and the abstract, and then the full text was obtained. All studies involving human participants or animals reporting on levels of total cholesterol, HDL, LDL, and triglycerides and the use of carob were included, and the findings were summarized and critically appraised.

### Lipid-Lowering Effects of Carob Extracts: Human Studies

As of 7 April 2022, there are 8 original studies on humans from 2001 to 2020 that observed the potential effects of carob on plasma lipid levels (Table 1). Most studies were randomized blinded cross-over studies or randomized, placebo-controlled, blinded parallel-arm clinical trials. Only one study looked at the difference between initial and final levels of parameters and was non-comparative.

**TABLE 1:** Human and animal studies on lipid-lowering effects of carob extracts.

No.	Author and date	Study description	Intervention	Follow up	Key findings	Overview of findings
1	Zunft et al., (2001)	Clinical trial (noncomparative, open-label study on hypercholesterolemic subjects)	Normal diet plus 15 g of carob preparation daily with meals such as a fruit muesli bar (36 g), powdered drink (35 g), or breakfast cereal (35 g). Each food product contained 5 g of a carob preparation (Caromaxtm^®^, Nutrinova, Germany). The products had to be taken immediately before or with breakfast, at lunch, and at dinner	8 w	Significant reduction in TC and LDL-C after 4 w, 6 w, and 8 w; maximal reduction in TC (7.8%) and LDL-C (12.2%) were observed after 6w; Significant reduction in LDL:HDL after 4 w; Except a small but significant reduction in HDL after 4 w, other changes in HDL and TAG were nonsignificant	↓TC ↓LDL-C ↓LDL:HDL
2	Zunft et al., (2003)	Randomized clinical trial (double-blind, placebo-controlled, and parallel arm study on hypercholesterolemic subjects)	The carob fiber group consumed 15 g of carob pulp preparation per day as an ingredient of 4 slices of bread (a total of 180 g; 2 slices in the morning, 2 slices in the evening) and one fruit bar (about 40 g) to be eaten at noon	Run-in phase of 2 w; intervention phase of 6 w	Significant reduction in TC (2.0 ± 1.8%), LDL-C (2.0 ± 1.8%), and a marginally significant reduction in LDL:HDL (7.9 ± 2.2%) in the carob group	↓TC ↓LDL-C ↓LDL:HDL
3	Gruendel et al., (2006)	Randomized crossover trial (on healthy subjects)	On the morning of every study day after a 10 h overnight fast, subjects consumed 400 ml of a standardized liquid meal (Pfrimmer-Nutricia^®^) within less than 5 min. Liquid meals were enriched with 0, 5, 10, or 20 g of carob fiber and provided in random order. The total fiber content was 74.6 g/100 g carob fiber corresponding to 68.4 g insoluble and 6.2 g soluble fiber. The total polyphenol content of the preparation was 2.8 g/100 g	Four sessions 300 min each, separated by intervals of 1w	Significant and dose-dependent reduction in TAG and serum NEFA at 60 min after a test meal consumption (*p* < 0.001)	Postprandially ↓TAG ↓NEFA
4	Gruendel et al. (2007)	Randomized crossover trial (on healthy subjects)	On the day preceding the blood sampling sessions (day 1), foods with or without a total of 50 g carob fiber were provided in a randomized order; The total fiber content was 4.6 g per 100 g carob fiber preparation corresponding to 68.4 g insoluble and 6.2 g soluble fiber. The content of water-soluble polyphenols in the preparation was 2.84 g per 100 g; the content of extractable polyphenols by organic solvents was 0.39 g per 100g; In the morning of study day 2, after a 10 h overnight fast, subjects consumed 103 g standardized white bread within 5 min	Two sessions 300 min each, separated by intervals of 1 w	Significant reduction in TAG (*p* = 0.033) and serum NEFA (*p* < 0.001) after a test meal consumption the day after	Postprandially ↓TAG ↓NEFA
5	Ruiz-Roso et al., (2010)	Randomized clinical trial (double-blind, placebo-controlled, and parallel arm clinical study on hypercholesterolemic subjects)	Subjects were asked to consume their usual diet with the addition of either, placebo or Exxenterol^®^. The dietary fiber used in this study was a concentrated polyphenols extract from carob (Exxenterol^®^, Puleva Biotech S.A.). This is a natural insoluble dietary fiber comprised of 80% insoluble polyphenols from carob pod	Run-in phase of 2 w; intervention phase of 4 w	Significant reduction in TC (17.8%), LDL-C (22.5%), LDL:HDL (26.2%), and TAG (16.3%) from baseline to post-intervention, compared to placebo; no significant changes in HDL-C. The effect of treatment with carob was greater in subjects with baseline levels higher than the average	↓TC ↓LDL-C ↓LDL:HDL ↓TAG
6	Banuls et al. (2016)	Clinical trial (randomized double-blind study on healthy subjects)	4.45 g (2.23 g, twice a day) of the Inositol enriched beverage (Fruit Up^®^, a commercially available product consisting of natural ingredients) or a sucrose-sweetened beverage, and maintained a normocaloric diet throughout the study. The composition of Fruit Up^®^ is as follows: naturally occurring soluble carbohydrates (monosaccharides, disaccharides, oligosaccharides, polyalcohols, and soluble fiber) and minor compounds (in trace contents: organic acids, minerals, amino-acids) derived principally from carob pods	run-in period of 1 m (normocaloric diet); 12 w	Significant reduction in Apo B levels and increase in LDL particle size at 12 weeks in the carob group	↓Apo-B ↑LDL particle size
7	Izaola et al., (2020)	Randomized clinical trial (double-blind, placebo-controlled, parallel-arm study on obese subjects)	50 g (two packages of 25 g per day) of snack enriched with wakame and carob pod flour	8 w	Significant reduction in TC (5.8%) (*p* = 0.02) and LDL-C (7.4%) (*p* = 0.03)	↓TC ↓LDL-C
8	Jaffari et al., (2020)	Quasi-experimental study on obese men	4 groups: resistance training, carob supplementation, combined training and supplementation, and control; participants consumed 1.5 g of carob seed powder in three capsules (500 mg) in three meals per day during 8 weeks	8 w	Significant reduction in TC (*p* = 0.001), LDL-C (*p* = 0.001), and TAG (*p* = 0.001), and increase in HDL-C (*p* = 0.001) in the resistance training group and a combination of resistance training and carob supplements group; carob supplements alone did not show a significant change	↓TC ↓LDL-C ↓TAG ↑HDL-C
9	Sanaa and Mohsen, (2006)	Male albino rats, sharls strain with diet-induced hyperlipidemia/hypercholesterolemia	Standard diet supplemented with 10% and 15% carob extract	6 w	Both groups of rats showed a decrease in the mean value of triglycerides, total cholesterol, LDL and VLDL and an increase in the HDL compared with positive control extract compared with positive control	↓TC ↓LDL-C ↓VLDL-C ↓TAG ↑HDL-C
10	Nassar, (2007)	Male Sprague-Dawley rats with diet-induced hyperlipidemia/hypercholesterolemia	6 w study: diets supplemented with carob molasses via drinking water (1% w/v) ad lib; Postprandial study: addition of 5% of carob molasses to lipid emulsion	6 w	After 6 weeks: significant increase in HDL-C in both high-fat and regular diet groups; no effect on TC, LDL, TAG, or apolipoprotein B; Postprandially: significant reduction in TAG, chylomicron-TAG, and chylomicron-cholesterol	After 6 w: ↑HDL-C Postprandially ↓TAG ↓CM-TAG ↓CM-C
11	Valero-Munoz et al., (2014)	Male New Zealand rabbits with diet-induced hyperlipidemia/hypercholesterolemia	Standard diet supplemented with carob pod supplement (1 g x kg-^1^ x d^-1^)	8 w	Significant reduction TC, LDL-C, and TAG in dyslipidemic group (*p* < 0.01)	↓TC ↓LDL-C ↓TAG
12	El-Manfaloty and Ali, (2014)	Male Sprague-Dawley rats with alloxan-induced diabetes	Standard diet supplemented with 10% and 20% carob extract	6 w	Significant reduction in TC, LDL-C, VLDL-C, and TAG and a significant increase in HDL-C in the 10% and 20% carob group compared to the dyslipidemic group	↓TC ↓LDL-C ↓VLDL-C ↓TAG ↑HDL-C
13	Hassanein et al. (2015)	Male Sprague-Dawley rats with diet-induced hyperlipidemia/hypercholesterolemia	Standard diet supplemented with 10% and 20% carob powder	6 w	Significant reduction in TC, LDL-C, VLDL-C, and TAG and a significant increase in HDL-C in the 10% and 20% carob group compared to the dyslipidemic group	↓TC ↓LDL-C ↓VLDL-C ↓TAG ↑HDL-C
14	Abu Hafsa et al., (2017)	Healthy male New Zealand rabbits fed standard diet.	Standard diet supplemented with 2.5%, 5%, 10% carob pod powder	120 d	Significant reduction in TC, LDL-C, HDL-C, and TAG (*p* < 0.05) in the carob group compared with the control group	↓TC ↓LDL-C ↓HDL-C ↓TAG
15	El Rabey et al., (2017)	Male rats (Rattus norvegicus of East China Origin) with diet-induced hyperlipidemia/hypercholesterolemia	Standard diet supplemented with 20% parsley seeds methanol extract and 20% carob legumes methanol extract	8 w	Significant reduction in TC, LDL-C, VLDL-C, and TAG and a significant increase in HDL-C in both parsley group and carob group compared to the hypercholesterolemic rats	↓TC ↓LDL-C ↓VLDL-C ↓TAG ↑HDL-C
16	Macho-Gonzalez et al., (2018)	Healthy male Wistar rats fed standard diet.	Standard diet supplemented with carob fruit pulp extract 25, 50, 150 mg/kg daily administrated by oral gavage	1 w; digestibility studies on the 1st d and 7th d	Postprandially: significant reduction in the TC and TAG after carob fruit pulp extract (25, 50, 150 mg/kg) treatments (all *p* < 0.001) in both study lengths	Postprandially ↓TC ↓TAG
17	Sour et al., (2019)	Male Wistar rats with diet-induced hyperlipidemia/hypercholesterolemia	Standard diet supplemented with 20% carob pulp extract	8 w	Significant reduction in TC, LDL-C, VLDL-C, and TAG in both obese and control groups; a significant increase in HDL-C only in obese rats	↓TAG ↓TC ↓VLDL-C ↓LDL-C ↑HDL-C
18	Valero-Munoz et al., (2019)	Male New Zealand rabbits with diet-induced hyperlipidemia/hypercholesterolemia	Standard diet supplemented with 3% of insoluble carob pod fiber	8 w	Significant reduction in TC and TAG in the dyslipidemic group treated with carob (*p* < 0.05)	↓TC ↓TAG
19	Macho-Gonzalez et al., (2019)	Male Wistar rats with streptozotocin-nicotinamide-induced diabetes and diet-induced hyperlipidemia/hypercholesterolemia	Standard diet supplemented with carob fruit extract (4 g/kg restructured meat) homogenized with lean mixed meat	8 w	Significant reduction in TC (*p* < 0.001), LDL-C (*p* < 0.001), VLDL-C (*p* < 0.001), IDL-C (*p* < 0.001), and TAG (*p* = 0.013); Significant increase in total mass of HDL (*p* < 0.001) in carob group compared to diabetic/hypercholesterolemic group	↓TC ↓LDL-C ↓VLDL-C ↓IDL-C ↓TAG ↑HDL total mass
20	De la Fuente-Fernandez et al., (2020)	C57/BL6J mice with diet-induced hyperlipidemia/hypercholesterolemia	Standard diet supplemented with 4.8% of CSAT+^®^ (carob pod and seed extract)	26 w	Significant reduction in TC (*p* < 0.05) and LDL-C (*p* < 0.05) in mice with metabolic syndrome treated with carob	↓TC ↓LDL-C
21	Macho-González et al., (2020)	Male Wistar rats with diet-induced diabetes and hyperlipidemia/hypercholesterolemia	Standard diet supplemented with carob fruit extract (4 g/kg restructured meat) homogenized with lean mixed meat	8 w	Significant reduction in VLDL-C (*p* = 0.005), IDL-C (*p* < 0.001), total mass of LDL (*p* = 0.003), and TAG (*p* = 0.017) in carob group compared to diabetic/hypercholesterolemic group	↓VLDL-C ↓IDL-C ↓LDL total mass ↓TAG
22	Rtibi et al., (2021)	Healthy male New Zealand rabbits fed standard diet.	Standard diet supplemented with 10 g/kg of Carob Powder and Whey powder or a mixture of 5 g/kg of Carob powder and 5 g/kg of Whey powder	7 w	Significant reduction in TC and TAG in carob group compared to control group	↓TC ↓TAG

TC, total cholesterol; LDL-C, LDL-cholesterol; VLDL-C, VLDL-cholesterol; IDL-C, IDL-cholesterol; HDL-C, HDL-cholesterol; TAG, triglycerides; NEFA, non-esterized fatty acid CM-TAG, chylomicron triglycerides; CM-C, chylomicron cholesterol; Apo-B, Apolipoprotein B; d, day(s); w, week(s); m, month(s).

Four studies found that carob pulp significantly reduced plasma levels of total cholesterol and LDL-cholesterol when it was used for a certain amount of time as a food supplement (Zunft et al., 2001; Zunft et al., 2003; Ruiz-Roso et al., 2010; Izaola et al., 2020). Two studies reported that carob also reduces LDL:HDL ratio (Zunft et al., 2001). However, in a 2003 study by Zunft et al., (2003) the LDL:HDL ratio was only marginally reduced. Reduction of TAG levels was not consistent: some found no significant changes (Zunft et al., 2001); some reported marginally significant changes (Zunft et al., 2003); finally, one study found a significant reduction (Ruiz-Roso et al., 2010). Levels of HDL were not significantly impacted by carob in all four studies (Zunft et al., 2001; Zunft et al., 2003; Ruiz-Roso et al., 2010; Izaola et al., 2020). Observable differences in results from these studies could be a consequence of many factors. Firstly, researchers used different carob products having a different compositions. Next, different doses of carob were used and treatment duration also varied. Also, in some studies subjects were hypercholesterolemic or obese, while other studies used healthy subjects. Finally, the duration of the follow-up period was also dissimilar. Additionally, some studies had a run-in phase while others did not. Regarding the efficacy of carob’s lipid-lowering activity, these studies found a decrease in LDL-C levels from 7.4% (Izaola et al., 2020) to 22.5% (Ruiz-Roso et al., 2010). In comparison, statin-associated reduction in LDL-C levels, being an agent and dose-dependent, results in a reduction in LDL-C ranging from <30% for low-intensity to ≥50% for high-intensity statin therapy (Grundy et al., 2019).

Banuls et al., (2016) found that carob resulted in a significant reduction in Apo-B levels while also significantly increasing the size of LDL particles. Although some theories suggest that small LDL particles have a greater atherogenic potential, studies have shown that both small and large LDL particles can cause atherosclerosis (Sacks and Campos, 2003). Moreover, the study included only healthy and not hypercholesterolemic subjects. Thus, it is not clear whether the same result would be seen in those subjects and to what extent.

Two studies analyzed whether the carob intake with meals has an impact on postprandial parameters. Both studies found significantly lower postprandial plasma levels of TAG and non-esterized fatty acids (Gruendel et al., 2006; Gruendel et al., 2007).

Results from a study by Jaffari et al.,(2020) showed that carob significantly lowers TAG, total cholesterol, LDL-cholesterol, and increases HDL-cholesterol levels only in participants that were involved in an exercise program. Carob alone did not have any effect on the lipid panel. This supports the well-known concept that exercise is an important lifestyle intervention for regulating lipid levels.

### Lipid-Lowering Effects of Carob Extracts: Animal Studies

As of 7 April 2022, there are 14 original studies on animals that investigated carob as a potential lipid-lowering agent (Table 1). Most studies were formulated so that there was a negative control group (normal diet), a positive control group (hypercholesterolemic group), and an experimental group (with carob included as a supplement). Most used were male Wistar rats, male Sprague-Dawley rats, or male New Zealand rabbits.

Eleven studies focused on measuring the effects of chronic (mostly several weeks) carob use on lipid profile. All eleven studies found that the carob supplementation significantly decreased total cholesterol, TAG, and LDL-cholesterol (Sanaa and Mohsen, 2006; Valero-Munoz et al., 2014; Hassanein et al., 2015; Abu Hafsa et al., 2017; El Rabey et al., 2017; Sour et al., 2019; Valero-Munoz et al., 2019; de la Fuente-Fernandez et al., 2020; Macho-González et al., 2020; Rtibi et al., 2021) and in some studies VLDL-cholesterol (Sanaa and Mohsen, 2006; Hassanein et al., 2015; El Rabey et al., 2017; Sour et al., 2019). Additionally, in contrast to human studies, five studies showed that HDL-cholesterol was significantly elevated after carob intake (Sanaa and Mohsen, 2006; Nassar, 2007; Hassanein et al., 2015; El Rabey et al., 2017; Sour et al., 2019). Like in the studies involving human participants, there are several limitations to the interpretation of these results together. Firstly, studies used different animal models. Secondly, the animals were fed with different carob preparations and different basal diets. Finally, study lengths were also variable.

El-Manfaloty et al. did a study on diabetic male Sprague-Dawley white albino rats with alloxan-induced diabetes. They found that both in healthy and diabetic animals, carob had a significant and positive impact on their lipid levels, namely on total cholesterol, TAG, LDL-cholesterol, VLDL-cholesterol, and HDL-cholesterol (El-Manfaloty and Ali, 2014). Similar results were observed by Macho-Gonzalez et al., (2019) who induced diabetes in male Wistar rats by intraperitoneal injection of streptozotocin and nicotinamide. The main difference between these two studies is that the study by El-Manfaloty et al. fed their experimental animals with a standard diet El-Manfaloty and Ali, 2014). On the other hand, Macho-Gonzalez et al., (2019) used a hyperlipidemic diet.

Nassar, (2007) and Macho-Gonzalez et al., (2018) studied an acute, postprandial response to carob supplementation. Both studies found that ingesting carob supplements after a meal significantly decreases plasma levels of TAG and total cholesterol.

## Discussion

The mechanism behind hypolipidemic effects of carob pulp is believed to be the synergistic action of its two key components: insoluble fiber and polyphenols. Several mechanisms have been proposed to explain this phenomenon, namely by affecting three organ systems: 1) gastrointestinal tract, 2) liver and 3) adipose tissue.

Gastrointestinal effects of carob pulp are: bile acid sequestration, digestive enzymes inhibition, delayed gastric emptying, and shortened intestinal transit time.

Many *in vivo* and *in vitro* studies have shown that carob can avidly bind bile salts and acids inside the intestinal lumen and consequently lead to two outcomes: decreased enterohepatic recirculation of bile acids and decreased absorption of cholesterol and fatty acids (Wursch, 1979; Zunft et al., 2001; Ruiz-Roso et al., 2010; El-Manfaloty and Ali, 2014; Valero-Munoz et al., 2014; Abu Hafsa et al., 2017; Macho-Gonzalez et al., 2018; Macho-Gonzalez et al., 2019; van Rijs and Fogliano, 2020). Impaired enterohepatic recirculation of bile acids subsequently leads to a higher rate of cholesterol conversion to bile acids which increases LDL uptake and de novo cholesterol synthesis inside hepatocytes (Zunft et al., 2003). Both fiber (Zunft et al., 2001; El-Manfaloty and Ali, 2014; Valero-Munoz et al., 2014; Abu Hafsa et al., 2017; Macho-Gonzalez et al., 2018; Macho-Gonzalez et al., 2019) and polyphenols (Wursch, 1979; Macho-Gonzalez et al., 2018; Macho-Gonzalez et al., 2019; van Rijs and Fogliano, 2020) are probably responsible for the above-mentioned mechanism. Polyphenols, namely tannins (Wursch, 1979; Abu Hafsa et al., 2017) and proanthocyanidins inhibit intestinal digestive enzymes, such as pancreatic lipase (Macho-Gonzalez et al., 2018). Furthermore, it has been proven that carob pulp causes delayed gastric emptying primarily by increasing the viscosity of gastric chyme due to the high insoluble fiber content. On the other hand, carob may also increase plasma levels of GLP-1 which is a known gastric motility inhibitor (Macho-Gonzalez et al., 2018). In addition, a study by Macho-Gonzalez et al.,(2020) showed that insoluble fibers and proanthocyanidins increase postprandial satiety and decrease food intake. It is also demonstrated that carob increases intestinal transit speed by trapping nutrients, enzymes, and bile acids inside the intestinal lumen (Macho-Gonzalez et al., 2018). This results in a greater volume of intestinal contents which stimulates bowel motility. As a result, the conjoined action of these mechanisms decreases triglycerides digestion and free fatty acids absorption. As some human and animal studies have shown (Gruendel et al., 2006; Gruendel et al., 2007; Nassar, 2007; Macho-Gonzalez et al., 2018), when given pre-prandially, carob reduced levels of TAG, NEFA, and/or total cholesterol. This, however, is just a temporary effect and its long-term consequences are yet to be determined.

Carob could also impact the hepatic metabolism directly. This can be further divided based on whether it alters cholesterol or triglycerides metabolism pathways.

A study by Zunft et al.,(2003) demonstrated that carob fiber caused a dose-dependent increase in the activity of cholesterol-7α-hydroxylase (Cyp7a), a bile acid synthesis enzyme. To increase cholesterol-to-bile acid conversion, plasma cholesterol or de novo synthesized cholesterol can be used. Proof of increased cholesterol de novo synthesis in the liver cells is the observed increased activity of HMG-CoA reductase caused by carob (Valero-Munoz et al., 2019).

Additionally, flavonoids, especially proanthocyanidins, increase the insulin levels which by acting on hepatocytes leads to a higher LDL-receptor density on the cell surface (Abu Hafsa et al., 2017; Macho-Gonzalez et al., 2019). Even though there is evidence of carob influence on these enzymatic and receptor systems, there is currently no certain way to determine if this is a primary or secondary effect of carob. Similar to bile acid sequestrating agents, like cholestyramine (Nazir et al., 1972), by reducing enterohepatic recirculation of bile acids, carob could ignite these pathways to allow the liver to produce more bile acids de novo. Further research needs to be conducted to evaluate these effects. Regarding triglyceride metabolism in hepatocytes, Valero-Muñoz et al. showed that carob had a significant impact on gene expression of various transcription factors and enzymes involved in this process (Valero-Munoz et al., 2014; Valero-Munoz et al., 2019). Specifically, it has been shown that carob increases the expression of sirtuin 1 (SIRT1), an enzyme that deacetylates transcription factors. Increased activity of SIRT1 leads to downregulation of sterol regulatory element-binding transcription factor 1c (SREBP-1c) and glycerol-3-phosphate acyltransferase (GPAT), both involved in triglyceride synthesis. Likewise, carob increases peroxisome proliferator-activated receptor gamma coactivator 1-alpha (PPARGC1A), also resulting in diminished intrahepatic lipogenesis.

Conjointly to the above-mentioned lipid-lowering mechanisms, carob is hypothesized to have a direct action on fatty tissue, as well.

Carob decreases free fatty acids released from the adipocytes (Macho-Gonzalez et al., 2019) which deprives the liver of triglycerides building blocks and subsequently renders the hepatocytes unable to secrete VLDL particles (Macho-Gonzalez et al., 2018).

In addition, polyphenols from carob contribute to a decrease in preadipocytes differentiation and proliferation while enhancing mature adipocytes’ apoptosis (Wang et al., 2014; Sour et al., 2019).

Apart from its hypolipidemic action, carob has also been considered to have antioxidative (El-Manfaloty and Ali, 2014; Abu Hafsa et al., 2017; Macho-Gonzalez et al., 2019; Sour et al., 2019; de la Fuente-Fernandez et al., 2020), anti-inflammatory and vascular-protective activity (Valero-Munoz et al., 2014; Valero-Munoz et al., 2019), as well as a positive impact on insulin resistance (Macho-González et al., 2020).

Concerning its antioxidative action, carob increases the activity of superoxide dismutase (SOD), catalase (CAT), and glutathione peroxidase (GPx) (Abu Hafsa et al., 2017). Moreover, polyphenols can scavenge reactive oxygen species (Sour et al., 2019). These effects are also considered to contribute to the hypolipidemic effect of carob.

In the study by Valero-Munoz et al., (2014) polyphenols and lower lipid levels showed substantial anti-inflammatory and vascular-protective action. They found an increase in acetylcholine-dependent vascular relaxation due to enhanced endothelial nitric oxide synthase (eNOS) gene expression. This study also found a decrease in atherosclerotic lesion area, and a decreased expression of transforming growth factor-beta (TGF- β), collagen 1, vascular cell adhesion molecule 1 (VCAM-1), CD36, tumor necrosis factor-alpha (TNF- α), and plasminogen activator inhibitor 1 (PAI-1). Through potentiation of the PI3K/AKT/mTOR pathway, carob improves insulin resistance (Macho-González et al., 2020).

These effects of carob, independent of its potential lipid-lowering activity, could be considered analogous to the pleiotropic effects of statins. However, studies that explored these effects of carob are all on animal models and these phenomena are yet to be determined in humans. Conversely, statin pleiotropic effects have been well documented in clinical trials (Oesterle et al., 2017).

In conclusion, carob fiber and polyphenols showed significant lipid-lowering effects, both in humans and animals through different mechanisms. All proposed mechanisms that explain the lipid-lowering activity of carob center around three main organ systems: 1) gastrointestinal tract, 2) liver and 3) adipose tissue. Also, carob products demonstrated antioxidative, anti-inflammatory, and vascular-protective activity in pre-clinical studies. However, even though these findings could seem promising, it must be taken into consideration that there are no confirmed effects of carob on meaningful outcomes, such as mortality. Therefore, it is important to note that statins remain the mainstay of dyslipidemia treatment as they have been shown to reduce the risk of major vascular events such as coronary events, coronary revascularization procedures, and stroke. Moreover, it has been proven that statins reduce cardiovascular mortality. With this in mind, carob supplementation could be considered as an addition to healthy lifestyle changes in mild cases of dyslipidemia or as an adjunct to standard hypolipidemic therapy. Finally, more studies with larger sample sizes should be done, especially on humans, to further investigate the potential use of carob extracts in the treatment of dyslipidemia. Also, further studies with isolated active principles from carob could reveal the potential candidate molecules thereby accelerating drug discovery.
